# LGAS-UNet: A Lightweight Network for Building Extraction from Remote Sensing Imagery in Complex Urban Scenes

**DOI:** 10.3390/s26185694

**Published:** 2026-09-08

**Authors:** Yaoyu Lu, Gang Cheng, Guosheng Cai, Baoquan Huang, Xiaonan Yu, Xinxu Guan, Xiangrong Fu

**Affiliations:** Key Laboratory of Spatio-Temporal Information and Ecological Restoration of Mines, Henan Polytechnic University, Ministry of Natural Resources of the People’s Republic of China, Jiaozuo 454003, China

**Keywords:** remote sensing imagery, complex urban environments, building extraction, lightweight network, attention mechanism

## Abstract

Accurate extraction of the spatial distribution of buildings from remote sensing imagery in complex urban environments is essential for urban planning and development. However, existing methods often suffer from high computational costs and insufficient building boundary recovery, making it difficult to achieve both efficient and accurate building extraction. To address these limitations, this study proposes LGAS-UNet, a lightweight network for building extraction. Based on the UNet architecture, LGAS-UNet replaces the original encoder with LSNet and incorporates a Global Context Aggregation Module (GCAM), Attention Gates (AGs), and Self-Calibrated Convolution (SCConv) modules into the encoder–decoder bridge, skip connections, and decoder feature-fusion units, respectively. These components enhance the global contextual representation of deep features, suppress irrelevant background responses during cross-level feature propagation, and improve feature fusion and boundary detail recovery during decoding. Experiments were conducted on the public WHU Building Dataset and a Zhengzhou building dataset constructed from satellite imagery. With only 6.12 M parameters and 3.64 G FLOPs, LGAS-UNet achieved an intersection over union (IoU) of 86.24%, an F_1_-score of 92.61%, and a boundary F_1_-score (BF-score) of 87.84% on the WHU dataset, achieving the best overall performance among the compared methods. On the Zhengzhou building dataset, LGAS-UNet achieved an IoU of 72.37%, an F_1_-score of 83.97%, and a BF-score of 74.09%, representing improvements of 1.70, 1.16, and 2.11 percentage points, respectively, over UNet. These results demonstrate that LGAS-UNet can efficiently and accurately extract buildings from remote sensing imagery in complex urban environments, providing a practical methodological reference for urban planning and management.

## 1. Introduction

Buildings constitute a fundamental component of urban spatial structures, and accurate information on their spatial distribution is essential for urban planning, disaster monitoring, and the continuous updating of geospatial information [1]. Consequently, the timely and accurate extraction of buildings from remote sensing imagery has become an important research topic in semantic segmentation [2]. Advances in high-resolution remote sensing technology have created an increasing demand for the efficient acquisition of building information. However, conventional field surveying and manual visual interpretation are constrained by high costs, low efficiency, and long update cycles, making them unsuitable for the rapid acquisition of building information over large areas. Accurate delineation of building boundaries remains particularly challenging in complex urban scenes characterized by interference from diverse land-cover types, substantial variations in building scale, and intricate boundary geometries [3]. Therefore, developing a building extraction model that effectively balances segmentation accuracy, boundary representation capability, and computational efficiency is of considerable importance for remote sensing applications in complex urban environments.

Early approaches to building extraction from remote sensing imagery primarily relied on conventional techniques, such as threshold-based segmentation, object-based image analysis, and ancillary information fusion. These methods generally identified buildings using manually designed spectral, textural, and geometric features [4,5,6]. Although effective in certain scenarios, they relied heavily on expert knowledge and handcrafted feature engineering. With advances in computing technology, conventional machine learning methods, including support vector machines (SVMs) and Markov random fields (MRFs), were gradually introduced into building extraction tasks, thereby increasing the degree of automation in the extraction process [7,8]. However, in complex urban environments, these methods remain vulnerable to background interference, substantial variations in building scale, and confusion between buildings and spectrally or visually similar land-cover classes. Consequently, they often fail to adequately capture high-level semantic information and fine-grained boundary details, leading to false-positive and false-negative predictions, as well as incomplete building boundaries [2]. Therefore, overcoming the limitations of traditional feature-driven approaches requires building extraction methods with stronger capabilities for feature learning and representation.

In the field of building extraction, subsequent studies have focused on improving multiscale feature modeling, adaptability to complex scenes, and boundary recovery. In recent years, deep learning has gradually become the predominant approach to building extraction from remote sensing imagery owing to its strong adaptive feature-learning capability. Long et al. proposed the Fully Convolutional Network (FCN), which enabled end-to-end pixel-wise prediction and promoted the transition of building extraction toward deep feature learning [9]. Subsequently, numerous deep learning-based building extraction methods have been developed. UNet employs an encoder–decoder architecture with skip connections to integrate low-level spatial details and high-level semantic information, thereby improving segmentation performance [10]. SegNet also adopts an encoder–decoder framework and progressively restores spatial resolution to achieve pixel-level segmentation [11]. Related dense prediction studies have also extended contextual and attention-based feature modeling to salient object detection in optical remote sensing imagery and landslide extraction from aerial imagery [12,13]. Similar strategies, including detail–semantic collaboration and visual attention-guided feature enhancement, have been applied to monocular depth estimation and concrete crack detection in complex scenes [14,15]. These studies further demonstrate the effectiveness of contextual modeling, detail preservation, and attention-guided feature selection in improving target discrimination and structural delineation under complex backgrounds. For example, Wang et al. integrated residual learning into UNet to enhance feature extraction [16]. Shao et al. proposed BRRNet by introducing a residual refinement module into UNet, thereby improving feature representation in complex building scenes [17]. DeepLabV3+ employs atrous spatial pyramid pooling to capture multiscale contextual information and combines it with a decoder to recover spatial details, yielding strong segmentation performance in building extraction tasks [18]. Hamaguchi et al. proposed a local feature extraction module with decreasing dilation rates to improve the segmentation of small and densely distributed objects in remote sensing imagery [19]. Qiu et al. incorporated an atrous spatial pyramid pooling module and improved depthwise separable convolution modules into the skip connections of UNet to enhance multiscale and multilevel feature representation [20]. Yang et al. proposed CSA-Net, a complex-scenario adaptive network that combines hierarchical contextual feature extraction, global–local feature interaction, and multiscale adaptive feature fusion. Although the network improved building extraction in complex scenes, its boundary recovery performance remained limited [21]. To improve computational efficiency, Paszke et al. proposed ENet, a lightweight semantic segmentation network specifically designed for low-latency applications. Its bottleneck structures and efficient convolutional operations substantially reduce the number of parameters and computational cost while retaining competitive segmentation accuracy [22]. Wu et al. developed CGNet, a lightweight context-guided network that jointly models local features and surrounding contextual information through context-guided modules while keeping model complexity low [23]. Sandler et al. introduced MobileNetV2, which employs inverted residual blocks and linear bottlenecks to reduce computational cost while preserving representational power [24]. More recently, Wang et al. proposed LSNet, which improves lightweight feature modeling to enhance representational capacity while reducing computational overhead [25]. However, when directly applied to building extraction, its lightweight design may provide insufficient representation of fine building boundaries and small-scale buildings, leaving room for improvement in extraction accuracy and cross-scene generalization.

Overall, although deep learning-based methods have achieved considerable progress in building extraction from remote sensing imagery, highly accurate models are often computationally intensive, whereas lightweight models may suffer from insufficient feature representation and degraded boundary details in complex urban scenes. To address the aforementioned challenges, this study proposes LGAS-UNet, a lightweight building extraction network designed for complex urban scenes. The proposed model employs LSNet as a lightweight encoder and integrates it with a UNet-style decoder, thereby maintaining a low parameter count and computational cost while preserving both high-level semantic information and low-level spatial details. To improve feature representation in complex scenes, a Global Context Aggregation Module (GCAM) is introduced at the encoder–decoder bridge to enhance the modeling of global contextual dependencies in deep features. Attention Gates (AGs) are incorporated into the skip connections to suppress irrelevant background responses during cross-level feature fusion [26], while Self-Calibrated Convolution (SCConv) modules are embedded in the decoder to improve multiscale feature fusion and boundary detail recovery [27]. In addition, the model is optimized using a composite loss function combining binary cross-entropy (BCE) loss and Dice loss to jointly optimize pixel-level classification and the structural completeness of building regions [28,29]. Experimental results on the WHU Building Dataset and the Zhengzhou Building Dataset demonstrate that the proposed method achieves strong overall performance in terms of building extraction accuracy, building completeness, and boundary recovery.

## 2. Materials and Methods

### 2.1. Architecture of LGAS-UNet

**Figure 1:**
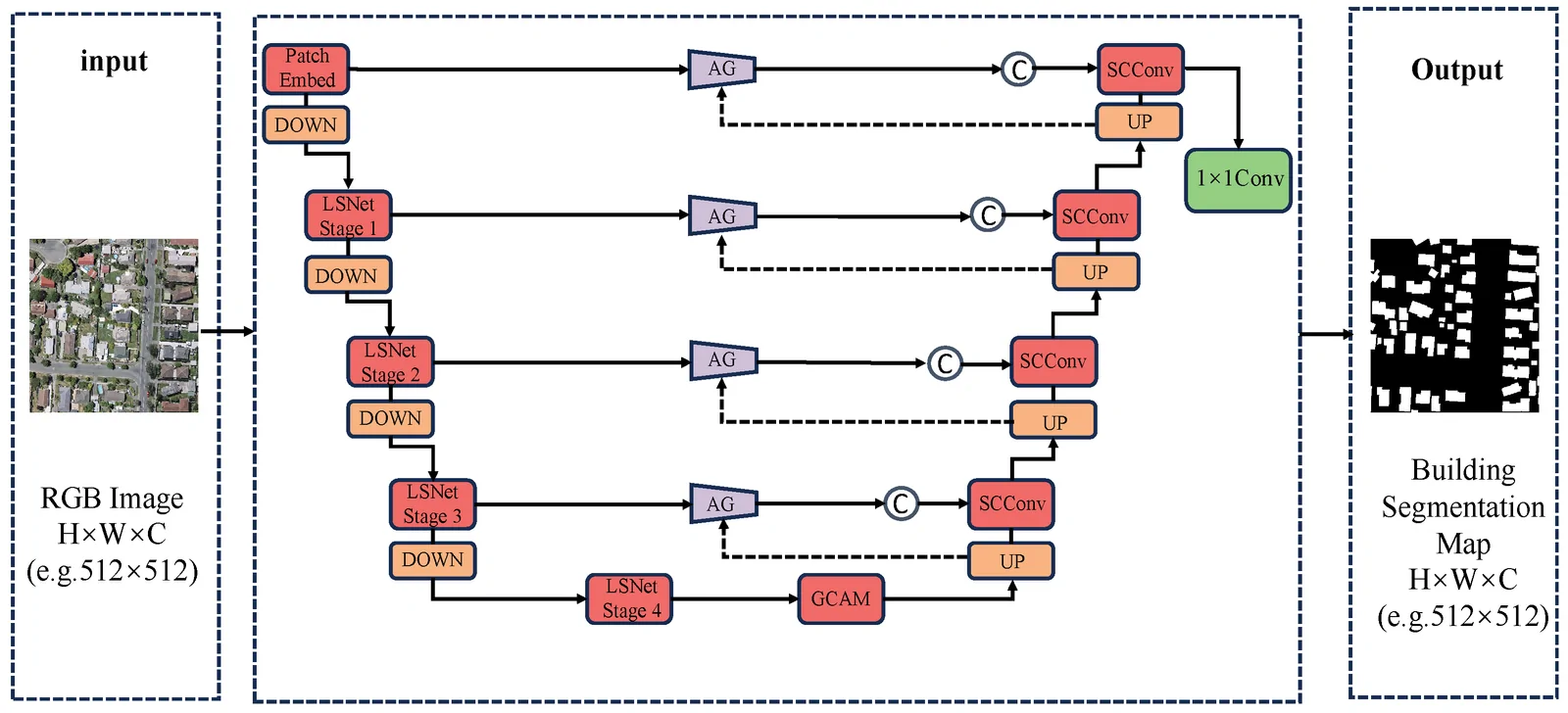
Overall architecture of LGAS-UNet.

To address the trade-off between lightweight design and segmentation accuracy in building extraction from high-resolution remote sensing imagery, this study proposes LGAS-UNet, a lightweight building extraction network whose overall architecture is illustrated in Figure 1. The proposed network employs LSNet as the encoder and a UNet-style decoder, enabling multilevel semantic feature extraction and progressive recovery of spatial details while maintaining a low parameter count and computational cost. To further improve feature representation in complex scenes, a GCAM is introduced at the encoder–decoder bridge to enhance global contextual modeling. AGs are embedded in the skip connections to suppress irrelevant background responses, while SCConv modules are incorporated into the decoder to improve multiscale feature fusion and boundary refinement.

**Figure 2:**
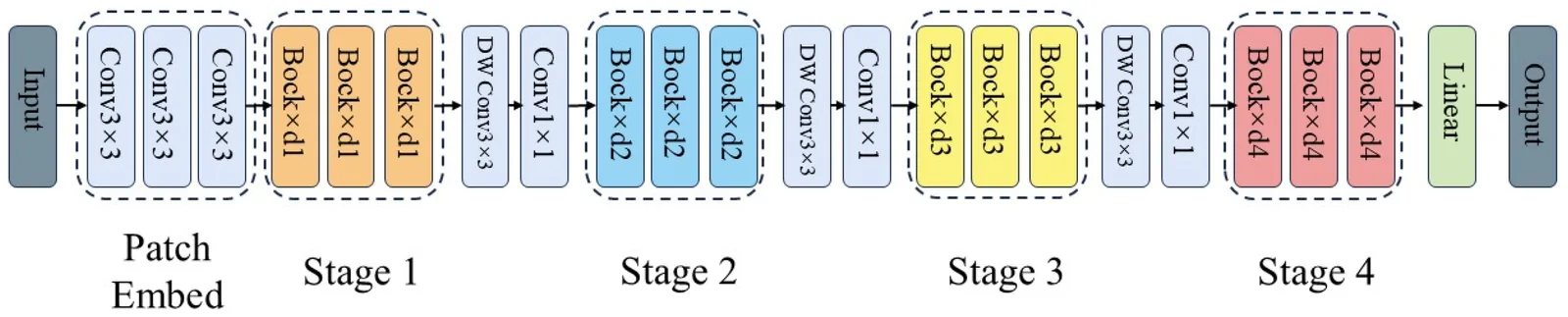
Architecture of the LSNet encoder.

During the encoding stage, LSNet serves as the lightweight backbone and hierarchically extracts features from the input remote sensing image to improve the network’s ability to distinguish buildings from complex backgrounds. The input image is first projected into an initial feature representation by the embedding layer and then progressively processed through multiple encoding stages. The encoder produces five feature maps at different spatial resolutions. The initial feature map primarily retains low-level spatial details, such as building edges and textures, whereas the progressively downsampled feature maps encode high-level semantic information at multiple scales. Together, these multilevel representations support subsequent global context modeling, feature selection in the skip connections, and feature reconstruction in the decoder. The architecture of the LSNet encoder is illustrated in Figure 2.

At the encoder–decoder bridge, the deepest encoder feature map is enhanced by the GCAM before being passed to the decoder, thereby strengthening high-level semantic representation. During decoding, building structures are gradually recovered through stagewise upsampling and skip connections. At each decoding stage, the encoder feature map at the corresponding spatial resolution is first adaptively weighted by an AG and then concatenated along the channel dimension with the decoder feature map upsampled to the same resolution. The concatenated features are subsequently processed by an SCConv module for cross-level feature fusion and refinement. This design improves the recovery of building structures and fine boundary details, thereby enhancing the structural completeness and boundary delineation accuracy of the segmentation results.

#### 2.1.1. Attention Gate

**Figure 3:**
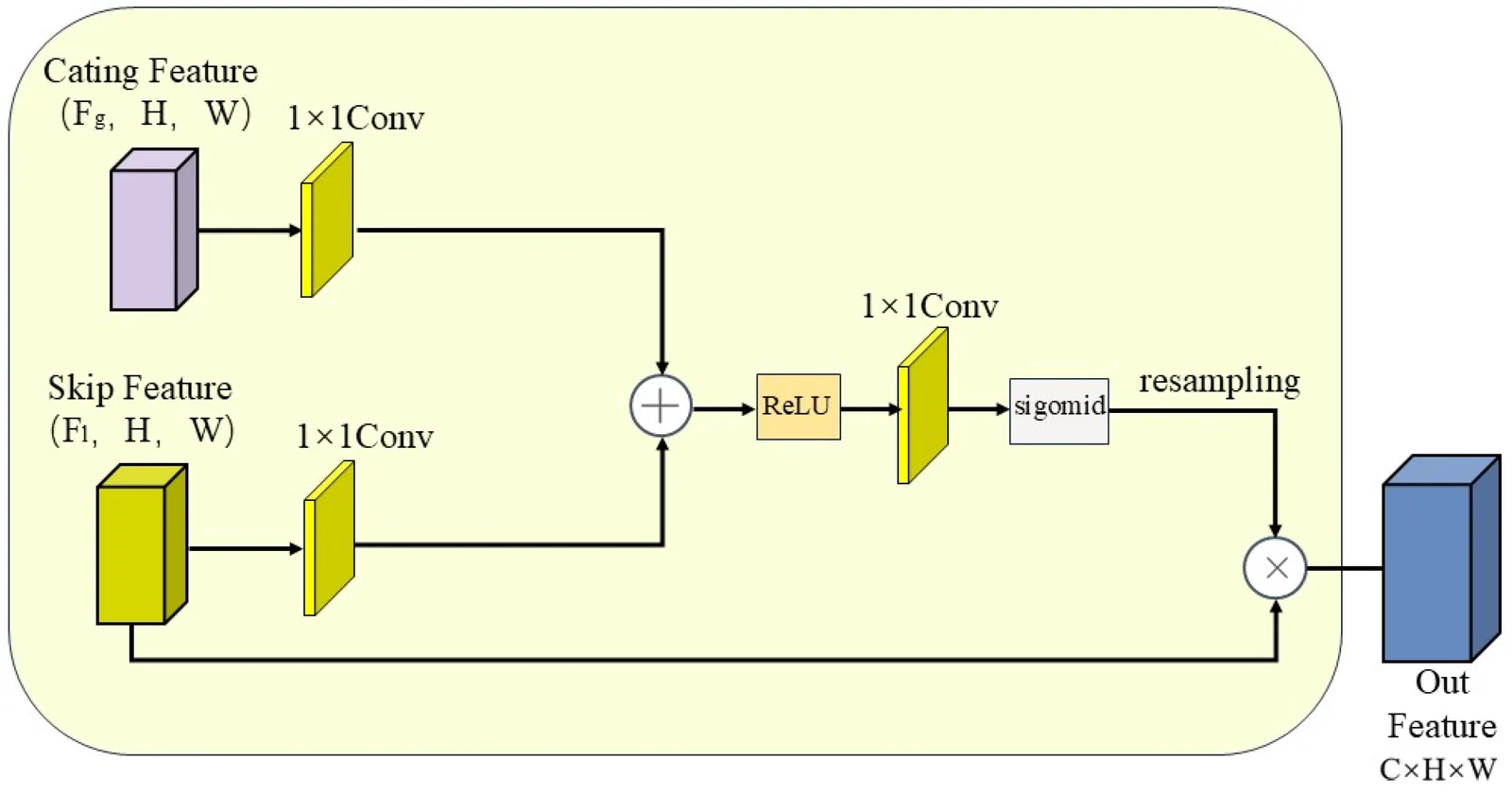
Architecture of the attention gate.

Although shallow encoder features retain rich edge and texture details, as well as precise spatial localization cues, their semantic discriminability is relatively limited. Consequently, skip connections may propagate irrelevant background responses together with building-related details, thereby interfering with cross-level feature fusion in the decoder [30]. In contrast, deeper decoder features possess stronger semantic discriminability but provide less precise spatial localization. Because the two feature types differ substantially in their semantic levels and feature distributions, directly concatenating them may introduce redundant background information into the decoder and reduce the effectiveness of feature fusion. To address this issue, an AG is incorporated into each skip connection, as illustrated in Figure 3. Before feature fusion, the upsampled decoder feature map, which carries high-level semantic information, serves as the gating signal to adaptively weight the detail-rich encoder feature map. This process enhances building-related responses while suppressing activations in non-target regions.

#### 2.1.2. Global Context Aggregation Module

**Figure 4:**
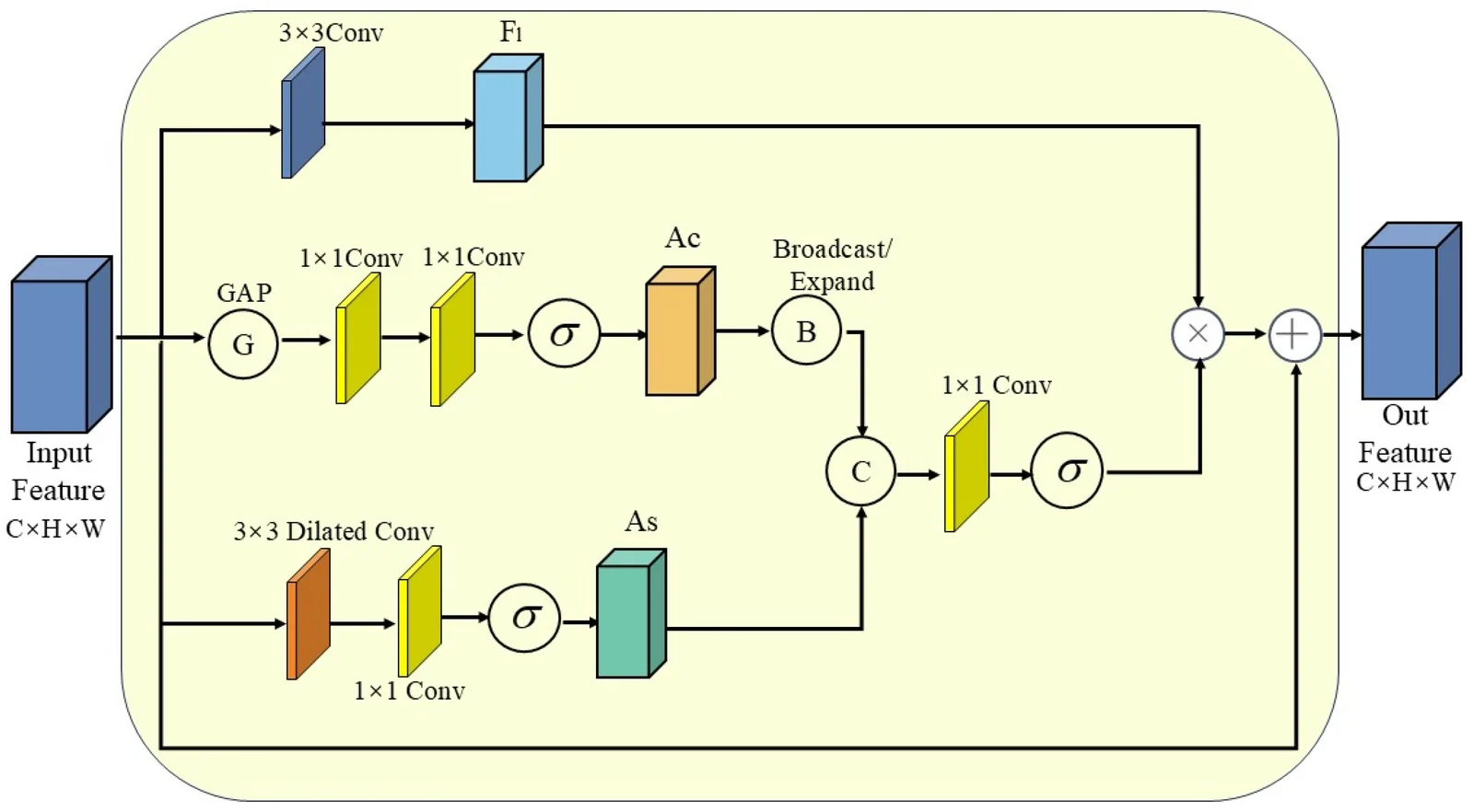
Architecture of the global context aggregation module.

Buildings in high-resolution remote sensing imagery exhibit substantial variations in scale and are often affected by interference from complex background elements, such as roads, vegetation, and shadows. Owing to the locality of convolutional receptive fields, deep features may fail to adequately capture overall building morphology and long-range spatial dependencies, thereby compromising the structural completeness of the extracted building regions. Previous studies have shown that channel attention mechanisms can improve feature selectivity by adaptively recalibrating channel responses [31,32], while large-kernel convolution can enlarge the effective receptive field and strengthen global spatial modeling [33]. To strengthen the network’s ability to represent global building structures, the GCAM is introduced at the encoder–decoder bridge of LGAS-UNet. By aggregating global contextual information, the GCAM enhances the global modeling and structural representation of deep semantic features. Its architecture is illustrated in Figure 4.

The channel attention branch of the GCAM adaptively recalibrates deep features along the channel dimension, enhancing responses in building-relevant semantic channels while mitigating interference from complex background elements, such as roads, vegetation, and shadows. This process improves the ability of the deep features to distinguish buildings from the background. Without introducing substantial parameter overhead, the branch selectively emphasizes informative channels, thereby satisfying the lightweight design objective of the network and producing a more discriminative feature representation for subsequent global context aggregation. The processing steps of the GCAM are described as follows:

(1) The input feature x is first passed through the channel attention branch to generate channel-wise attention weights. These weights are then applied to the original input feature to obtain the channel-recalibrated feature y, expressed as

(1) $$y=x⊙M_{C}(x)$$

where *Mc*(*x*) denotes the channel attention mapping function, and *θ* denotes element-wise multiplication.

(2) The recalibrated feature y is projected into an embedding space, in which the pairwise similarities between spatial locations are computed to construct a global affinity matrix A. The affinity matrix is subsequently used to perform globally weighted feature aggregation, thereby capturing long-range contextual dependencies within building regions.

(3) Finally, the globally aggregated contextual feature is combined with the original input feature through a residual connection. This operation preserves the original semantic information while producing the enhanced output feature:

(2) $$F_{out}=x+W_{0}(Ag(y))$$

where *A* denotes the global affinity matrix, *g*(*y*) denotes a linear feature transformation, *w*_0_ denotes the output projection, and *F_out_* represents the output feature of the GCAM.

Through these operations, the GCAM preserves the original deep semantic information while strengthening global structural coherence and contextual representation within building regions. This enhanced representation helps reduce the erroneous merging of adjacent buildings, fragmented building predictions, and spatial discontinuities in local feature responses under complex background conditions, thereby providing more informative features for boundary recovery and fine-grained segmentation in the subsequent decoding stages.

#### 2.1.3. Self-Calibrated Convolution Module

**Figure 5:**
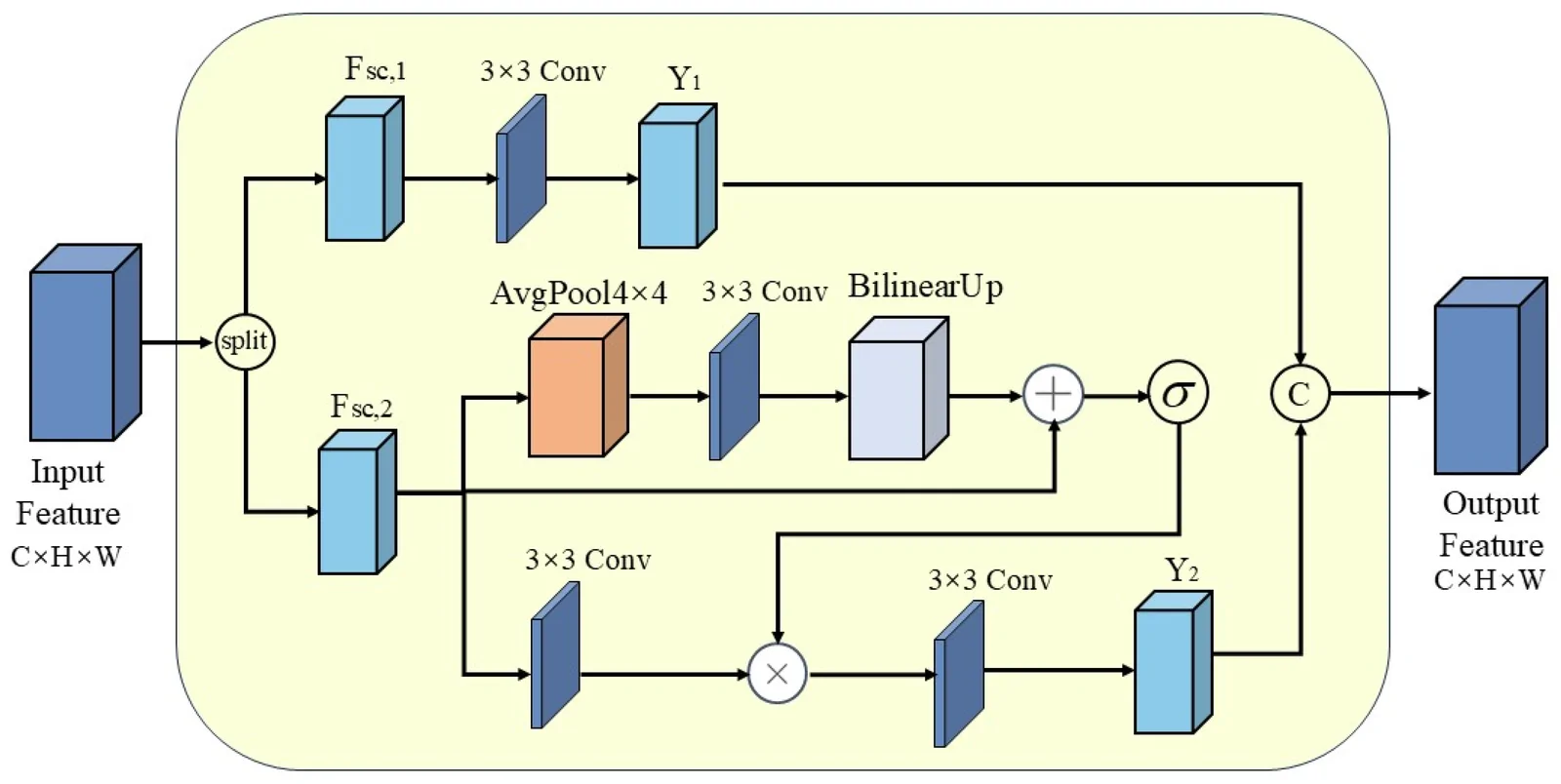
Architecture of the self-calibrated convolution module.

Nested encoder–decoder architectures, such as UNet++, improve multilevel feature integration through redesigned skip pathways [34]. Nevertheless, shallow encoder features and deep semantic features still differ in their semantic levels and response patterns. Consequently, conventional convolution may be insufficient to effectively capture local contextual information and recover fine boundary details from the concatenated representations. To enhance the spatial feature calibration and contextual representation of the fused features, SCConv modules are embedded in the decoder feature-fusion units to adaptively recalibrate and refine the fused representations [27]. The architecture of the SCConv module is illustrated in Figure 5.

SCConv dynamically adjusts feature responses by introducing a multiscale contextual calibration branch into local convolutional modeling. Specifically, the input feature map is split into two channel groups, which are processed by two parallel branches. The first branch applies conventional convolution to extract local spatial information, whereas the second branch enlarges the receptive field through downsampling and aggregates neighborhood contextual information at a reduced spatial resolution. After being upsampled to the original spatial resolution, the contextual feature map is used to generate self-calibration weights that adaptively modulate the feature responses in the corresponding branch. Finally, the calibrated feature map is concatenated with the output of the local branch along the channel dimension, producing an output representation that preserves local details while incorporating contextual information.

### 2.2. Loss Function

Building extraction from remote sensing imagery is often affected by a substantial class imbalance between building and background pixels. To achieve stable pixel-level optimization while imposing region-level structural constraints, a composite loss function combining binary cross-entropy (BCE) loss and Dice loss is employed to provide joint supervision at both the pixel and region levels. The two component losses and the total loss are defined as follows:

(3) $$L_{CE}=-\frac{1}{N}\sum_{i=1}^{N}\left[y_{i}logp_{i}+(1-y_{i})log(1-p_{i})\right]$$

(4) $$L_{Dice}=1-\frac{2\sum_{i=1}^{N}p_{i}y_{i}+\varepsilon }{\sum_{i=1}^{N}p_{i}+\sum_{i=1}^{N}y_{i}+\varepsilon }$$

(5) $$L_{total}=\lambda L_{CE}+\left(1-\lambda \right)L_{Dice}$$

In Equations (3)–(5), N denotes the total number of pixels included in the loss computation, *yi* ∈ {0, 1} denotes the ground-truth label of the *i*-th pixel, and *Pi* ∈ {0, 1} denotes the predicted probability that the ith pixel belongs to the building class. The parameter ε is a smoothing constant introduced to ensure numerical stability, and the weighting coefficient *λ* is set to 0.5. By jointly optimizing the BCE and Dice losses, the proposed model promotes accurate pixel-wise classification while strengthening region-level constraints on building completeness and complex boundary structures.

### 2.3. Experimental Setup

#### 2.3.1. Datasets

**Figure 6:**
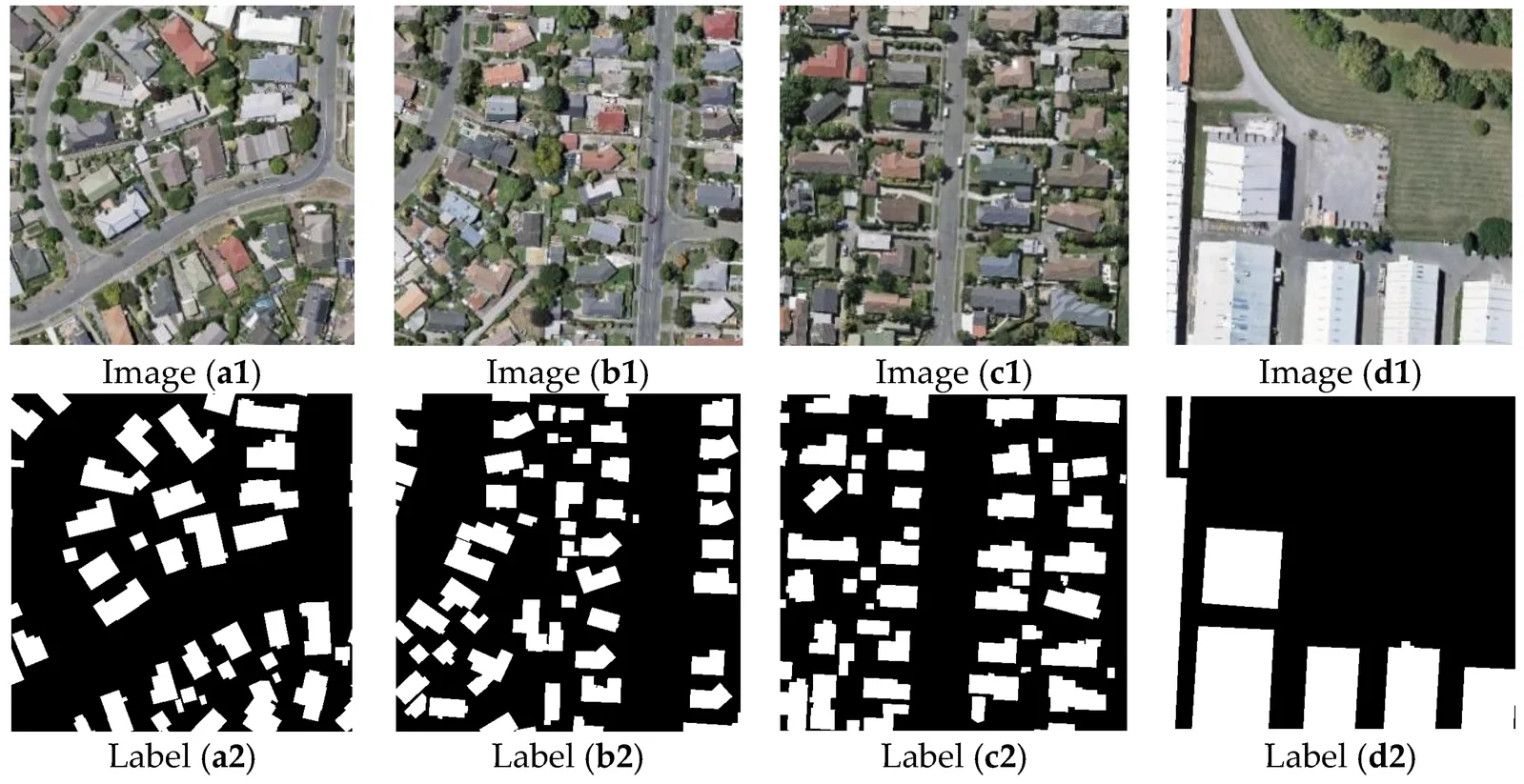
Representative image–label pairs from the WHU Building Dataset: (**a1**–**d1**) remote sensing images; (**a2**–**d2**) corresponding ground-truth labels.

To evaluate the effectiveness and applicability of the proposed method, experiments were conducted on the publicly available WHU Building Dataset and the Zhengzhou Building Dataset developed in this study. Released by Wuhan University, the WHU Building Dataset [35] covers approximately 450 km^2^ in Christchurch, New Zealand, and contains more than 220,000 individual building footprints. The dataset comprises 8188 aerial images, each with dimensions of 512 × 512 pixels and a spatial resolution of 0.3 m. To ensure comparability with previous studies, the official data split was adopted, consisting of 4736 training images, 1036 validation images, and 2416 test images. Representative image–label pairs from the dataset are shown in Figure 6.

**Figure 7:**
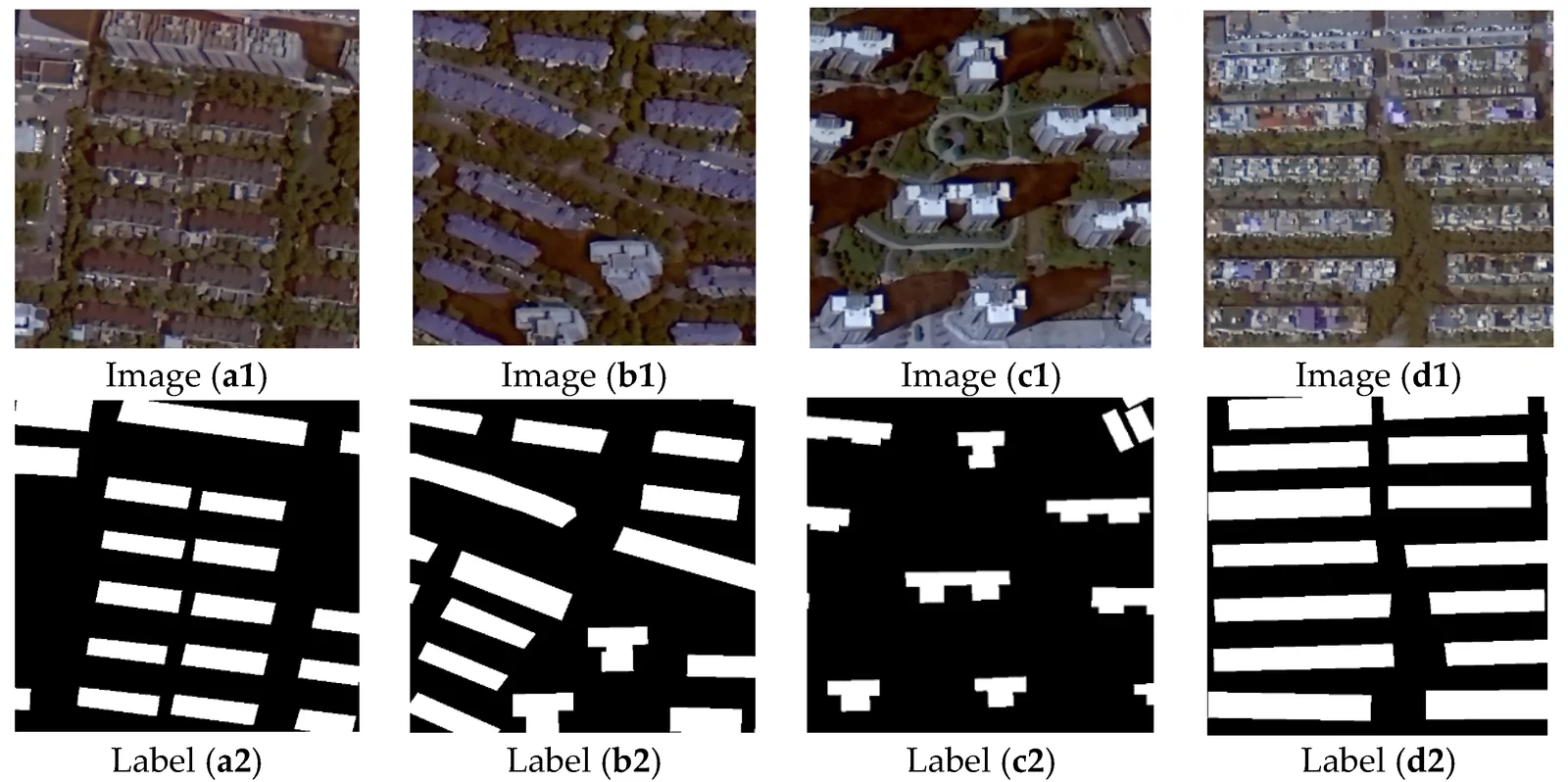
Representative image–label pairs from the Zhengzhou Building Dataset: (**a1**–**d1**) remote sensing images; (**a2**–**d2**) corresponding ground-truth labels.

To evaluate the applicability of the proposed method to complex, densely built urban scenes, the Zhengzhou Building Dataset was developed using Jilin-1 satellite imagery. Experienced remote sensing interpreters manually delineated building footprints with reference to multitemporal high-resolution historical imagery. Applying sliding-window cropping before dataset splitting can result in spatially adjacent and highly overlapping patches being assigned to different subsets, leading to data leakage. To avoid this issue, the source imagery was first divided into training and validation subsets at the original-image level. Patches of 512 × 512 pixels were then generated independently within each subset using a sliding window with an overlap ratio of 0.5. The resulting dataset comprises 2754 training patches and 627 validation patches. Representative image–label pairs from the dataset are shown in Figure 7.

#### 2.3.2. Implementation Details

The experiments were conducted using the PyTorch deep learning framework on a workstation equipped with an NVIDIA GeForce RTX 5060 GPU with 8 GB of memory. The operating system was Windows 11, and the software environment included Python 3.10.20 and PyTorch 2.11.0 with CUDA 13.0 support. All models were trained and evaluated under the same hardware and software configurations to ensure a fair comparison. During training, the input image size was set to 512 × 512 pixels, the batch size was set to 2, and the number of training epochs was set to 100. The Adam optimizer was adopted with an initial learning rate of 0.0001.

#### 2.3.3. Evaluation Metrics

To comprehensively evaluate the building extraction performance of the proposed model, *Recall*, *F_1_-score*, and Intersection over Union (*IoU*) are employed as region-level evaluation metrics, while the Boundary *F_1_-score* (*BF-score*) is used to assess boundary quality. *Recall* measures the proportion of ground-truth building pixels correctly identified by the model, whereas the *F_1_-score* reflects the balance between precision and recall. *IoU* quantifies the regional overlap between the predicted segmentation mask and the ground-truth mask. The *BF-score* evaluates the localization of building contours and the recovery of fine boundary details based on boundary matching. Together, these metrics provide a comprehensive assessment of regional overlap, building completeness, and boundary delineation accuracy. They are calculated as follows:

(6) $$R=\frac{N_{TP}}{N_{TP}+N_{FN}}$$

(7) $$F_{1}=\frac{2N_{TP}}{2N_{TP}+N_{FP}+N_{FN}}$$

(8) $$IoU=\frac{N_{TP}}{N_{TP}+N_{FP}+N_{FN}}$$

(9) $$BFscore=\frac{2\times Precision_{edge}\times Recall_{edge}}{Precision_{edge}+Recall_{edge}}$$

In Equations (6)–(9), *N_TP_* denotes the number of building pixels correctly classified as buildings, *N_FP_* denotes the number of non-building pixels incorrectly classified as buildings, and *N_FN_* denotes the number of building pixels incorrectly classified as non-building. *P_edge_* and *R_edge_* denote boundary precision and boundary recall, respectively. Higher values of these metrics indicate better performance in terms of building extraction accuracy, structural completeness, and boundary recovery.

## 3. Results

To evaluate the effectiveness of the proposed method, the experimental results are analyzed from three perspectives: ablation studies, comparative experiments, and efficiency analysis. Specifically, the ablation studies assess the individual contributions and synergistic effects of the key components; the comparative experiments evaluate the overall building extraction performance of the model across different datasets and urban scenes; and the efficiency analysis examines the trade-off between segmentation accuracy and computational cost.

### 3.1. Ablation Studies

Ablation studies were conducted on both the WHU Building Dataset and the Zhengzhou Building Dataset to evaluate the effectiveness of the individual components. As shown in Table 1, the original UNet achieved an IoU of 83.40%, an F_1_-score of 90.95%, and a BF-score of 84.95% on the WHU Building Dataset. Replacing the original encoder with LSNet resulted in the lightweight LSNet-UNet baseline, which achieved an IoU of 81.92%, an F_1_-score of 90.06%, and a BF-score of 83.34%. These results indicate that replacing the original encoder with LSNet led to a moderate decline in baseline segmentation performance. After incorporating the GCAM into LSNet-UNet, the IoU, F_1_-score, and BF-score increased to 83.78%, 91.17%, and 85.27%, respectively, demonstrating that global context modeling improves the overall representation of building regions. When the AGs were introduced independently, the model achieved an IoU of 82.72%, a recall of 88.27%, an F_1_-score of 90.54%, and a BF-score of 84.46%. In particular, recall decreased by 1.69 percentage points relative to the LSNet-UNet baseline, indicating that attention-based feature selection improved several region- and boundary-level metrics but may also have suppressed some building responses, resulting in reduced recall. Incorporating SCConv alone produced an IoU of 82.07%, an F_1_-score of 90.15%, and a BF-score of 84.71%, indicating modest improvements in decoder feature fusion and boundary quality. Among the two-component configurations, combining the GCAM with the AGs yielded an IoU of 84.60% and an F_1_-score of 91.66%, whereas combining the GCAM with SCConv produced a BF-score of 86.18%. These results suggest that the components provide complementary benefits for region segmentation and boundary recovery. When the GCAM, AGs, and SCConv were incorporated simultaneously, the full LGAS-UNet model achieved an IoU of 86.24%, an F_1_-score of 92.61%, and a BF-score of 87.84%, with all three metrics reaching their highest values among the evaluated configurations. Relative to the LSNet-UNet baseline, these metrics increased by 4.32, 2.55, and 4.50 percentage points, respectively; relative to the original UNet, the corresponding gains were 2.84, 1.66, and 2.89 percentage points. Overall, enhancing the LSNet-UNet baseline through global context modeling at the encoder–decoder bridge, feature selection in the skip connections, and feature refinement in the decoder effectively compensates for the performance loss caused by replacing the original encoder with LSNet and further improves building extraction and boundary recovery.

As shown in Table 2, the LSNet-UNet baseline without the GCAM, AGs, or SCConv modules achieved an IoU of 68.94%, an F_1_-score of 81.61%, and a BF-score of 69.12% on the Zhengzhou Building Dataset, all of which were lower than those obtained by the original UNet. Introducing each component individually improved model performance to varying degrees. In particular, incorporating the GCAM increased the IoU, F_1_-score, and BF-score to 70.74%, 82.86%, and 71.26%, respectively. The two-component configurations further improved the overall performance of the LSNet-UNet baseline. Combining the GCAM with the AGs yielded an IoU of 71.35% and an F_1_-score of 83.28%, whereas combining the GCAM with the SCConv modules produced a BF-score of 72.46%. When the GCAM, AGs, and SCConv modules were incorporated simultaneously, the full LGAS-UNet model achieved an IoU of 72.37%, an F_1_-score of 83.97%, and a BF-score of 74.09%, with all three metrics reaching their highest values among the evaluated configurations. Relative to the LSNet-UNet baseline, these metrics increased by 3.43, 2.36, and 4.97 percentage points, respectively. These results demonstrate that introducing targeted feature-enhancement mechanisms at the encoder–decoder bridge, in the skip connections, and within the decoder feature-fusion units effectively improves region-level segmentation and boundary extraction performance.

### 3.2. Quantitative Comparison

To evaluate the segmentation performance of the proposed model, LGAS-UNet was compared with UNet, DeepLabV3+, SegNet, ENet, LMFFNet, and CGNet. As shown in Table 3, LGAS-UNet achieved an IoU of 86.24%, a recall of 91.78%, an F_1_-score of 92.61%, and a BF-score of 87.84% on the WHU Building Dataset, outperforming all competing methods on all four metrics. Compared with SegNet, LGAS-UNet increased the IoU, recall, F_1_-score, and BF-score by 1.06, 1.13, 0.62, and 1.72 percentage points, respectively. Relative to the lightweight model LMFFNet, the corresponding gains were 3.56, 2.70, 2.09, and 5.37 percentage points. These results indicate that LGAS-UNet captures discriminative features of building regions more effectively, thereby improving the accuracy and completeness of building extraction while also enhancing boundary localization and contour recovery.

As shown in Table 4, LGAS-UNet achieved an IoU of 72.37%, a recall of 84.89%, an F_1_-score of 83.97%, and a BF-score of 74.09% on the Zhengzhou Building Dataset. Although its recall was 0.43 percentage points lower than that of ENet, LGAS-UNet achieved the highest IoU, F_1_-score, and BF-score among all evaluated methods, indicating strong overall building extraction performance on this dataset. Compared with SegNet, LGAS-UNet increased the IoU, recall, F_1_-score, and BF-score by 1.42, 2.08, 0.96, and 2.21 percentage points, respectively. Relative to the lightweight model LMFFNet, the corresponding gains were 1.66, 0.15, 1.13, and 1.93 percentage points. These results show that LGAS-UNet performs effectively in scenes characterized by densely distributed buildings, complex backgrounds, and substantial background clutter. It improves region-level segmentation accuracy while enhancing building boundary localization and contour recovery.

To visually compare the ability of different models to recover spatial details, boundary structures, and overall building morphology, representative scenes were selected for qualitative analysis. As shown in Rows 1–3 of Table 5, conventional fully convolutional and lightweight networks, such as UNet and ENet, exhibited pronounced boundary erosion and extensive missed detections in densely built scenes. Although DeepLabV3+ alleviated some missed detections, noticeable merging persisted in the narrow gaps between adjacent small buildings. In contrast, LGAS-UNet substantially reduced missed detections while better preserving the separation and topological independence of individual building footprints. The yellow-boxed region in Row 4 further highlights the model’s ability to delineate a shadow-affected corner of a large building. All competing methods exhibited severe boundary collapse at this right-angle corner, whereas LGAS-UNet more accurately reconstructed its geometric boundary.

To further evaluate the adaptability and stability of the proposed model in complex satellite remote sensing scenes, a qualitative comparison of the segmentation results on the Zhengzhou Building Dataset was conducted. Table 6 presents the visual extraction results obtained by the compared models. Compared with the aerial imagery in the WHU Building Dataset, the Zhengzhou scenes contain more densely distributed buildings and are more severely affected by surrounding vegetation and shadows. As shown in Groups 1 and 2, which contain dense clusters of elongated buildings, UNet, SegNet, and ENet exhibited extensive internal missed detections within elongated building regions, together with noticeable merging in the narrow gaps between adjacent buildings. These errors may partly result from the limited ability of local convolutional operations to capture long-range structural dependencies. Although DeepLabV3+ improved the internal completeness of some buildings through atrous convolution, boundary erosion remained evident. In contrast, LGAS-UNet better preserved the structural continuity and topological independence of elongated buildings. This behavior is consistent with the role of the GCAM in modeling long-range dependencies and providing large-scale spatial constraints for densely distributed buildings with elongated shapes. Groups 3 and 4 highlight building boundaries severely affected by shadows from surrounding objects. In these low-contrast regions, LMFFNet [36] and CGNet exhibited noticeable missed detections of small buildings and contraction of geometric boundaries. By contrast, LGAS-UNet more completely extracted weak-response building regions affected by shadows and produced smoother and more regular boundaries. Overall, the qualitative results suggest that the proposed model provides greater stability and robustness for building extraction in complex urban scenes.

### 3.3. Model Efficiency Comparison

For building extraction from remote sensing imagery, a model should not only achieve high segmentation accuracy but also support efficient processing and deployment in large-scale applications. Therefore, the balance between extraction accuracy and computational efficiency is a key consideration when evaluating the practical applicability of a model. Increasing network depth or introducing complex feature-modeling modules can enhance semantic representation; however, these strategies generally increase the number of parameters and computational cost, thereby limiting deployment efficiency in large-area remote sensing applications. To further evaluate the overall performance of different models, we conducted a quantitative comparison of model parameters, computational complexity, inference speed, and FPS on the WHU dataset under the same hardware environment and experimental settings. The results are presented in Table 7.

As shown in Table 7, conventional networks such as UNet and SegNet required substantially greater computational resources and achieved relatively low training throughput, which may limit their efficient deployment for large-scale remote sensing image processing. Lightweight networks such as CGNet and ENet achieved higher computational efficiency, but this advantage was accompanied by lower extraction accuracy; for example, CGNet achieved an IoU of 78.97%. The proposed LGAS-UNet achieves a favorable balance between computational efficiency and segmentation accuracy. The model contains only 6.12 M parameters and requires 3.64 G FLOPs, which accounts for only 2.2% of the computational cost of UNet. Meanwhile, it achieves an inference speed of 144.59 FPS, demonstrating that the introduced GCAM, AG, and SCConv modules enhance feature representation capability without introducing significant inference overhead.

## 4. Discussion

LGAS-UNet achieved favorable segmentation performance on both the WHU Building Dataset and the Zhengzhou Building Dataset. These results suggest that the proposed model performed effectively on two datasets representing different imaging conditions and urban scenes. Compared with conventional segmentation networks, LGAS-UNet achieved higher IoU, F_1_-score, and BF-score values with a relatively low parameter count and computational cost. Relative to existing lightweight networks, it better preserved the completeness of extracted building regions and recovered building boundaries. These findings indicate that reducing network complexity alone may weaken semantic representation and detail recovery. Accordingly, incorporating targeted global context modeling, cross-level feature selection, and boundary refinement into a lightweight backbone provides an effective approach to improving the performance of lightweight building extraction models.

The ablation results further clarify the contributions of the proposed components. Introducing the GCAM, AGs, or SCConv modules individually improved the IoU, F_1_-score, and BF-score to varying degrees. The three components enhance building extraction through global context modeling at the encoder–decoder bridge, cross-level feature selection in the skip connections, and feature refinement within the decoder feature-fusion units, respectively. The two-component configurations generally produced greater performance gains than the corresponding single-component configurations, while the simultaneous integration of the GCAM, AGs, and SCConv modules yielded the best overall results on both datasets. Therefore, the improvements achieved by LGAS-UNet cannot be attributed to a single component alone but instead appear to arise from the complementary effects of the three components. Their joint use enables the model to retain its lightweight design while improving the completeness of building regions and the recovery of boundary details.

The results obtained on the two datasets reveal the performance variations of LGAS-UNet under different imaging conditions and urban environments. On the WHU Building Dataset, LGAS-UNet achieves superior regional segmentation accuracy and boundary quality, demonstrating its effectiveness in handling diverse building scales and locally ambiguous boundaries. In contrast, the overall performance of the evaluated models decreases on the Zhengzhou Building Dataset, which can be mainly attributed to the lower spatial resolution of satellite imagery, dense building distributions, shadow occlusion, and complex background interference. Nevertheless, LGAS-UNet consistently maintains competitive performance in terms of IoU, F1-score, and BF-score, indicating its effectiveness in extracting buildings under challenging urban scenarios. It should be noted that the current experiments are conducted under independent training and testing settings for each dataset, and cross-dataset evaluation has not been performed to comprehensively assess the generalization capability of LGAS-UNet under variations in geographic regions, sensor characteristics, and data distributions. Since remote sensing datasets usually exhibit differences in imaging conditions, spatial resolutions, urban morphology, and annotation criteria, the proposed model may still suffer from domain shift when applied to unseen scenarios. Future research will focus on investigating the transferability of lightweight building extraction models across different regions and sensors, as well as exploring domain adaptation strategies to further improve their generalization capability.

From the perspective of the accuracy–efficiency trade-off, LGAS-UNet achieves a favorable balance between model complexity and segmentation performance. Conventional segmentation networks commonly rely on relatively large parameter counts and high computational costs to strengthen semantic representation. Although such networks can achieve competitive segmentation performance, their computational demands may limit rapid processing and deployment over large remote sensing areas. Lightweight networks provide greater computational efficiency but may suffer from insufficient semantic representation, degraded boundary details, and missed detections of small buildings. By combining the lightweight LSNet encoder with low-overhead feature-enhancement components, LGAS-UNet improves region-level segmentation accuracy and boundary recovery while maintaining low computational complexity. The proposed model therefore shows practical potential for efficient building extraction from large-area remote sensing imagery. Although pruning and quantization techniques can further reduce model size and computational cost, this study mainly focuses on achieving lightweight design through network architecture optimization. The proposed LGAS-UNet improves computational efficiency by designing an efficient encoder and introducing lightweight feature enhancement modules, rather than relying on post-training compression strategies. Nevertheless, pruning and quantization methods may provide additional optimization opportunities for practical deployment. Future work will investigate the combination of architectural optimization and model compression techniques to further improve the efficiency and deployment capability of lightweight building extraction networks.

## 5. Conclusions

To address the difficulty of balancing extraction accuracy and computational complexity in building extraction from remote sensing imagery, this study proposes an efficient lightweight network termed LGAS-UNet. By integrating the GCAM, AGs, and SCConv modules, the proposed network coordinates global context modeling, the suppression of background noise during cross-level feature transfer, and the reconstruction of fine spatial geometry. Experimental results on the WHU Building Dataset and the Zhengzhou Building Dataset demonstrate that LGAS-UNet achieves a favorable balance between segmentation accuracy and computational efficiency. The model contains 6.12 million parameters and requires 3.64 G FLOPs, corresponding to approximately 2.26% of the FLOPs required by UNet. On the WHU Building Dataset, LGAS-UNet achieved an IoU of 86.24% and an F_1_-score of 92.61%, demonstrating that it maintains effective building extraction performance at relatively low computational cost. Nevertheless, its ability to separate adjacent individual buildings in densely built scenes remains limited. Future work will focus on improving the separation of individual buildings in dense urban areas and exploring the joint optimization of super-resolution reconstruction and building extraction to improve the recovery of boundary details from lower-resolution imagery.

## Figures and Tables

**Table 1:** Ablation results on the WHU Building Dataset.

Variant	Model	IoU (%)	Recall (%)	F_1_ (%)	BF-Score (%)
1	UNet	83.40	90.19	90.95	84.95
2	LSNet-UNet	81.92	89.96	90.06	83.34
3	LSNet-UNet + GCAM	83.78	89.78	91.17	85.27
4	LSNet-UNet + AG	82.72	88.27	90.54	84.46
5	LSNet-UNet + SCConv	82.07	90.01	90.15	84.71
6	LSNet-UNet + GCAM + AG	84.60	90.36	91.66	86.11
7	LSNet-UNet + AG + SCConv	83.52	89.19	91.02	85.42
8	LSNet-UNet + GCAM + SCConv	84.25	90.30	91.45	86.18
9	LGAS-UNet	86.24	91.78	92.61	87.84

**Table 2:** Ablation results on the Zhengzhou Building Dataset.

Variant	Model	IoU (%)	Recall (%)	F_1_ (%)	BF-Score (%)
1	UNet	70.67	83.35	82.81	71.98
2	LSNet-UNet	68.94	82.63	81.61	69.12
3	LSNet-UNet + GCAM	70.74	83.51	82.86	71.26
4	LSNet-UNet + AG	69.42	84.18	81.95	70.34
5	LSNet-UNet + SCConv	69.77	83.26	82.19	70.81
6	LSNet-UNet + GCAM + AG	71.35	84.28	83.28	72.18
7	LSNet-UNet + AG + SCConv	70.52	82.74	82.71	71.57
8	LSNet-UNet + GCAM + SCConv	71.15	83.90	83.14	72.46
9	LGAS-UNet	72.37	84.89	83.97	74.09

**Table 3:** Quantitative comparison of different models on the WHU Building Dataset.

Variant	Model	IoU (%)	Recall (%)	F_1_ (%)	BF-Score (%)
1	UNet	83.40	90.19	90.95	84.95
2	DeepLabV3+	84.77	90.52	91.75	85.73
3	SegNet	85.18	90.65	91.99	86.12
4	ENet	81.93	88.93	90.07	82.01
5	LMFFNet	82.68	89.08	90.52	82.47
6	CGNet	78.97	87.75	88.25	79.36
7	LGAS-UNet	86.24	91.78	92.61	87.84

**Table 4:** Quantitative comparison of different models on the Zhengzhou Building Dataset.

Variant	Model	IoU (%)	Recall (%)	F_1_ (%)	BF-Score (%)
1	UNet	70.67	83.35	82.81	71.98
2	DeepLabV3+	70.84	84.44	82.93	72.41
3	SegNet	70.95	82.81	83.01	71.88
4	ENet	69.78	85.32	82.20	70.36
5	LMFFNet	70.71	84.74	82.84	72.16
6	CGNet	68.83	84.51	81.54	69.28
7	LGAS-UNet	72.37	84.89	83.97	74.09

**Table 5:** Visualization of segmentation results on the WHU Building Dataset.

Group	Image	Label	UNet	SegNet	DeepLabV3+	ENet	LMFFNet	CGNet	LGAS-UNet
1	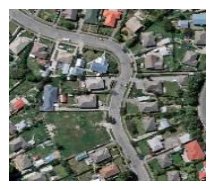	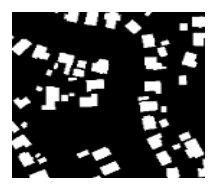	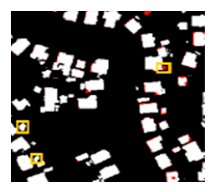	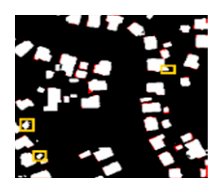	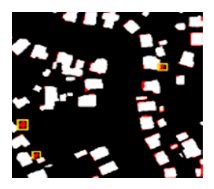	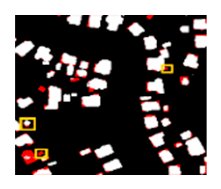	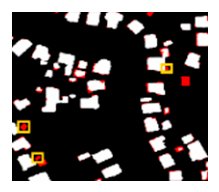	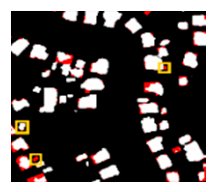	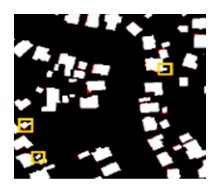
2	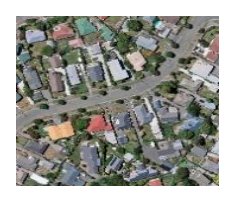	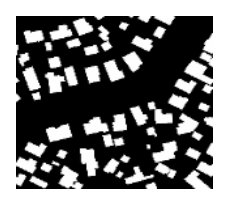	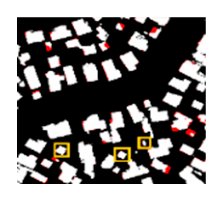	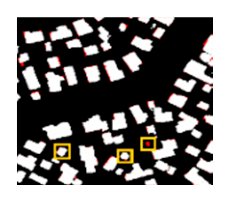	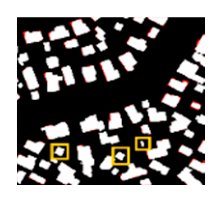	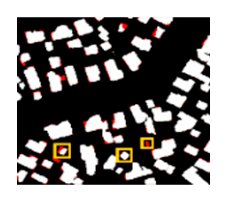	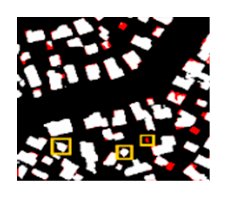	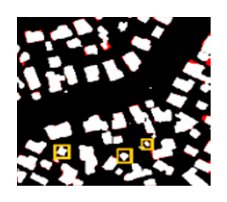	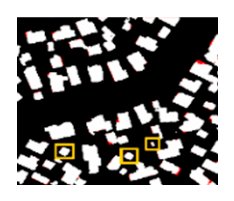
3	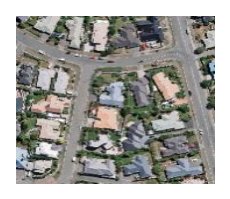	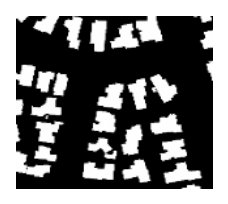	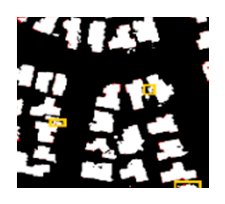	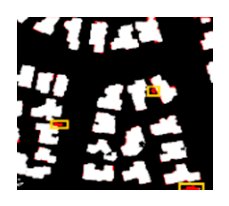	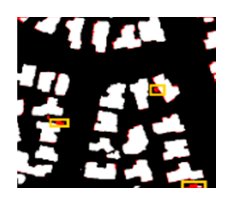	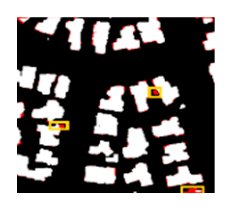	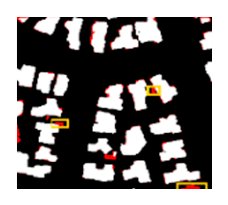	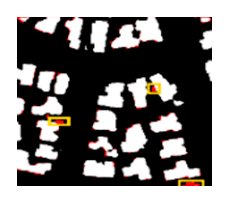	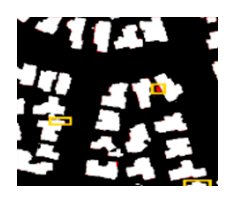
4	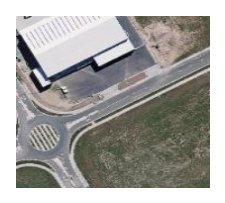	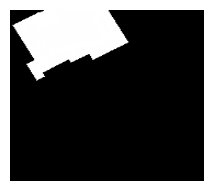	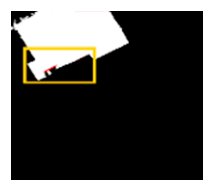	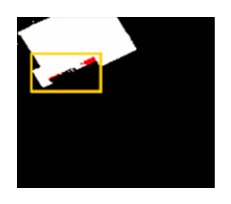	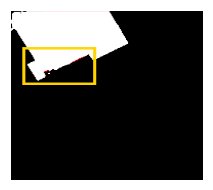	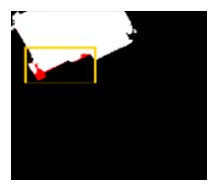	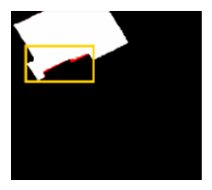	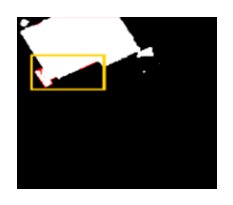	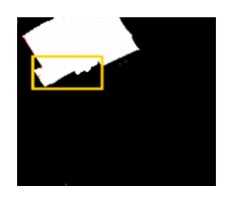
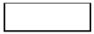 Building 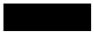 Non-building 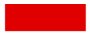 Missed detection 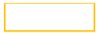 Highlighted region									

**Table 6:** Visualization of segmentation results on the Zhengzhou Building Dataset.

Group	Image	Label	UNet	SegNet	DeepLabV3+	ENet	LMFFNet	CGNet	LGAS-UNet
1	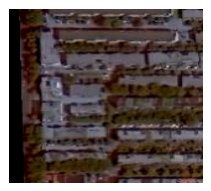	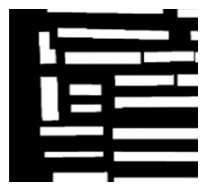	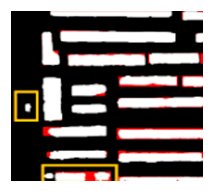	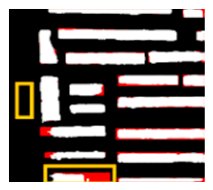	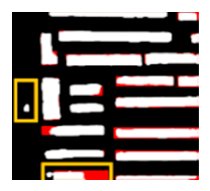	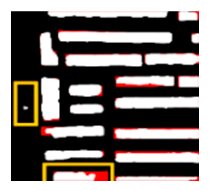	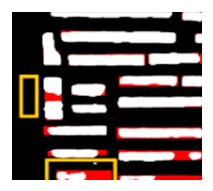	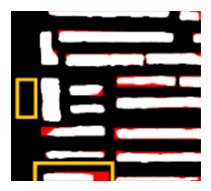	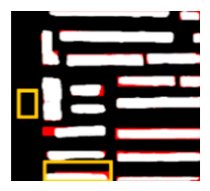
2	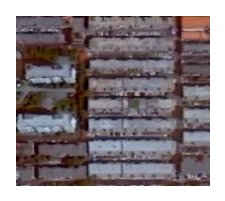	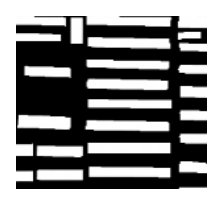	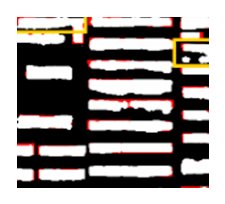	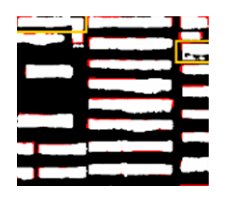	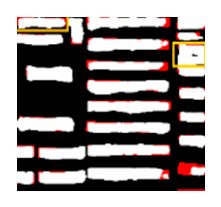	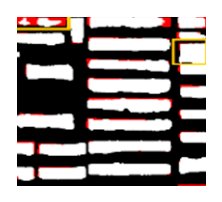	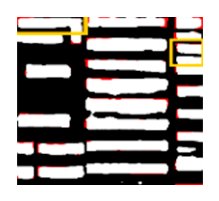	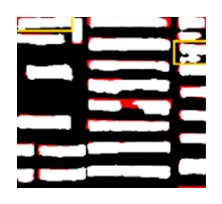	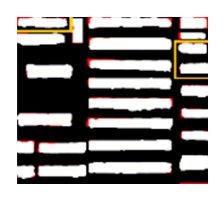
3	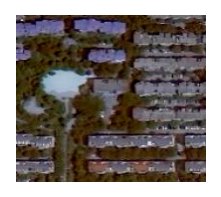	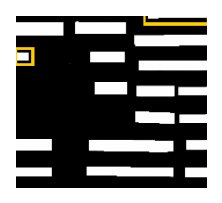	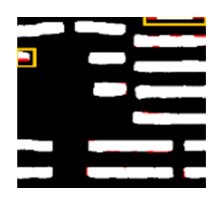	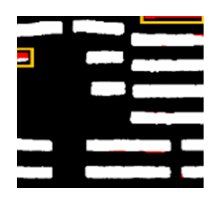	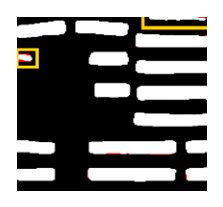	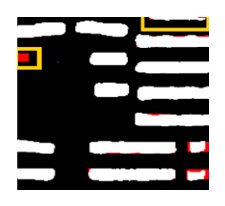	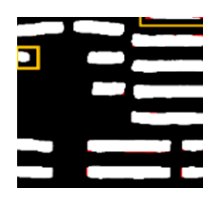	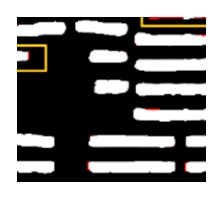	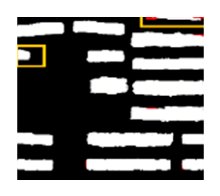
4	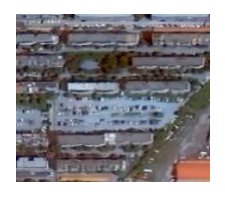	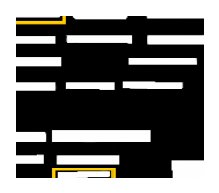	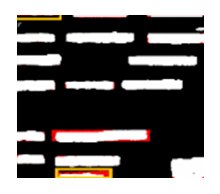	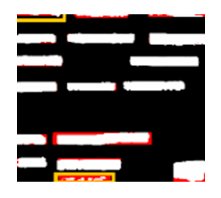	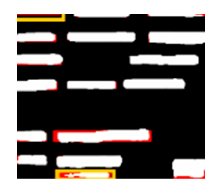	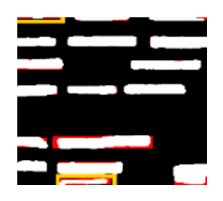	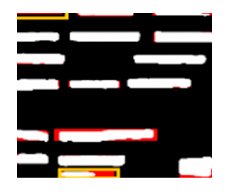	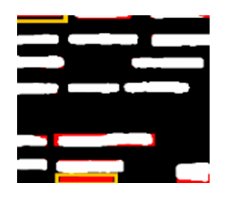	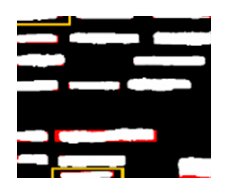
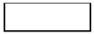 Building 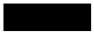 Non-building 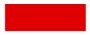 Missed detection 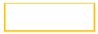 Highlighted region									

**Table 7:** Performance and efficiency comparison on the WHU Building Dataset.

Model	Params (M)	FLOPs (G)	Speed (Batch/s)	Time (h)	IoU (%)	F_1_ (%)	FPS
UNet	17.26	160.76	4.37	15.11	83.40	90.95	22.67
DeepLabV3+	40.35	69.45	9.77	7.12	84.77	91.75	48.79
SegNet	29.44	133.36	5.12	12.94	85.18	91.99	30.47
ENet	0.36	2.18	21.74	3.14	81.93	90.07	167.34
LMFFNet	1.34	8.31	16.82	4.05	82.68	90.52	130.26
CGNet	0.49	3.55	19.99	3.57	78.97	88.25	152.15
LGAS-UNet	6.12	3.64	19.33	3.82	86.24	92.61	144.59

## Data Availability

The raw data supporting the conclusions of this article will be made available by the authors on request.
